# Adverse Skeletal Muscle Adaptations in Individuals Born Preterm—A Comprehensive Review

**DOI:** 10.3390/cimb46050276

**Published:** 2024-05-10

**Authors:** Nick L. Dobson, Danielle E. Levitt, Hui Ying Luk, Heather L. Vellers

**Affiliations:** 1 Independent Researcher, Oklahoma City, OK 73162, USA; lepageou39@gmail.com; 2 Department of Kinesiology and Sport Management, Texas Tech University, Lubbock, TX 79409, USA

**Keywords:** preterm birth, skeletal muscle hypertrophy, mitochondrial dysfunction, inflammation

## Abstract

Infants born preterm face an increased risk of deleterious effects on lung and brain health that can significantly alter long-term function and quality of life and even lead to death. Moreover, preterm birth is also associated with a heightened risk of diabetes and obesity later in life, leading to an increased risk of all-cause mortality in young adults born prematurely. While these preterm-birth-related conditions have been well characterized, less is known about the long-term effects of preterm birth on skeletal muscle health and, specifically, an individual’s skeletal muscle hypertrophic potential later in life. In this review, we discuss how a confluence of potentially interrelated and self-perpetuating elements associated with preterm birth might converge on anabolic and catabolic pathways to ultimately blunt skeletal muscle hypertrophy, identifying critical areas for future research.

## 1. Introduction

Worldwide, an estimated 15 million infants are delivered preterm each year [1], and preterm birth (PB) increases the risk for multiple adverse health conditions throughout an individual’s life. Prematurity is categorized based on gestational age (extremely preterm, <28 weeks; very preterm, 28–32 weeks; moderate preterm, 32–34 weeks; late preterm, 34–36 weeks), and the magnitude of prematurity reflects disease propensity. Overall, infants born preterm carry a greater risk of bronchopulmonary dysplasia [2], brain injury [3], and oxygen toxicity [4] than infants born at full term (≥37 weeks of gestation). Morphological heart and vasculature abnormalities can adversely alter cardiac function in infants born preterm, with the severity of the impairment being closely tied to low gestational birth weight (<2500 g) [5]. Skeletal muscle function [6] and motor control [7,8,9] also correlate with birth weight and gestational age in young children. Indeed, low body weight at birth correlates with compromised motor skill development in young children born preterm [7,8,9], while gestational age is positively correlated with physical fitness in children (3–6 years of age) born preterm [6]. Moreover, adults born prematurely carry an increased risk for cardiometabolic and cardiovascular disease [10,11,12,13], poor body composition (i.e., high body fat percentage) [14], poor muscular strength and endurance [15], and a potentially blunted hypertrophic response to resistance exercise [16,17,18]. Whether directly or indirectly, these factors can ultimately lead to impaired overall skeletal muscle function into adulthood.

While PB is associated with a host of cardiometabolic conditions and shortened life expectancy [19], many of these are preventable and treatable for most individuals with lifestyle interventions, including diet and exercise. However, individuals born prematurely (especially those born at or earlier than 32 weeks’ gestation) do not respond well to exercise training [20,21,22]. Multiple studies have found that PB adversely affects the growth of the heart, whereby the ventricular size and overall heart mass are smaller [13,23,24,25] among young adults relative to those born at full term. These works also show that PB-associated heart abnormalities contribute to an impaired exercise response. Similar to PB-induced effects on the heart, there are also known adverse impacts on skeletal muscle. Individuals born preterm show blunted insulin sensitivity [26], low circulating vitamin D [27] and insulin-like growth factor-1 (IGF-1) concentrations [28], and a relatively higher proportion of type II muscle fibers [29] than control counterparts. Further, other findings show that prematurity is associated with higher levels of oxidative stress [30], potential epigenetic changes [31], and potential alterations to skeletal muscle amino acid uptake [17]. While each of these factors has the potential to impair muscle growth and development, they could also mitigate resistance-training-induced gains that might otherwise offset some of these limitations, potentially leading to an increased risk of metabolic diseases such as type 2 diabetes and obesity—conditions that are highly prevalent in adults born preterm. This review will highlight these factors and discuss how they collectively alter skeletal muscle function in individuals born prematurely.

## 2. Abnormal Insulin Signaling and Low IGF Levels

PB is becoming increasingly recognized as a risk factor for both childhood- and adult-onset type I and II diabetes, with studies showing a 1.2–2.5-fold greater risk in individuals born preterm than those born at full term [17,32,33]. The relationship between PB and type II diabetes in both children and adults is thought to be related to very low birth weight (<1500 g), which could confer reduced insulin sensitivity because of PB-related reductions in muscle mass [34,35]. More broadly, numerous other studies have shown that there is a positive correlation between muscle mass and insulin sensitivity [36,37,38]. For example, Kalyani et al. [37] analyzed data from 587 participants (aged 26–95 years) and found that high fasting glucose and insulin levels correspond to low midthigh muscle mass. Additionally, using a cross-sectional design that included 14,807 adult participants (aged 18–65 years), Kim and Park [39] showed that a high-muscle/low-fat phenotype corresponds to high insulin sensitivity, while a high-fat/low-muscle phenotype corresponds to low insulin sensitivity. These results support the notion that PB-related reductions in insulin sensitivity and corresponding diabetes risk are, at least in part, associated with low muscle mass.

In addition to low muscle mass at birth, infants born preterm also carry a heightened risk of vitamin D deficiency since vitamin D is transferred from the mother to the fetus during the third trimester [27]. Moreover, some evidence suggests that PB-related reductions in serum vitamin D concentrations can persist into adolescence. Indeed, Wood et al. [27] showed that 26% (12/46) of adolescents (aged 12–18 years) born preterm displayed either vitamin D deficiency or insufficiency. The authors also found that serum vitamin D concentration positively correlates with insulin sensitivity, which is unsurprising given that vitamin D deficiency can cause poor insulin sensitivity (via decreases in insulin receptor expression) and impaired β-cell function [27,40,41]. Vitamin D supplementation has therefore been proposed as a possible strategy for treating poor insulin sensitivity—a common condition among adults born preterm [26]. However, the efficacy of this treatment strategy remains uncertain. Yousefi Rad et al. [42] showed that 100 microgram doses of vitamin D significantly decrease HbA1c and fasting insulin in type II diabetic patients, while a meta-analysis that included 12 studies and a total of 1181 overweight subjects found no significant effect of vitamin D supplementation on measures of fasting blood glucose and insulin [43]. The results of these studies suggest that further work is likely needed to draw definitive conclusions regarding the efficacy of vitamin D supplementation in improving insulin sensitivity. Interestingly, Chen et al. [44] showed that muscle protein synthesis increases following vitamin D supplementation when combined with resistance training and whey protein supplementation in a cohort of 58 healthy, untrained males aged 18–24 years. While these results are encouraging and demonstrate a potential link between vitamin D availability and insulin-mediated increases in muscle protein synthesis, the effect of vitamin D supplementation on skeletal muscle hypertrophy is not known in individuals born prematurely who engage in resistance training.

While insulin’s contribution to muscle protein synthesis appears to be moderated by amino acid uptake and muscle perfusion [45,46], it is known that insulin stimulates the protein kinase B (Akt)/mammalian target of rapamycin complex 1 (mTORC1) pathway and, therefore, increases muscle protein synthesis in a perfusion- and amino acid availability-dependent manner [46,47]. Thus, individuals suffering from either type I or II diabetes demonstrate impairments in insulin secretion and/or poor glucose uptake that hinder the anabolic effects of insulin on skeletal muscle tissue [48]. It is important to note that skeletal muscle anabolic potential depends on numerous factors, including an optimal hormonal milieu [49]; therefore, anabolic hormones represent only a portion of the factors that influence skeletal muscle hypertrophy. Regardless, insulin is a powerful anti-catabolic hormone, and alterations to insulin secretion or sensitivity can potentially promote muscle atrophy in individuals born preterm, given their predisposition to vitamin D deficiency [27,40,41,50] and low muscle mass [34,35,51].

Circulating levels of IGF-1 are lower in PB infants than in those born at full term [52,53,54]. Moreover, some studies have identified very low birth weights (<1500 g) and concomitant low levels of circulating IGF-1 as factors that limit children’s ability to reach their genetic height potential [53] and lead to a shorter stature regardless of size for gestational age (i.e., small or average) [55]. Notably, while fetal tissues have detectable levels of IGF-1 during the first trimester, the most significant increase in circulating IGF-1 occurs in the third trimester [54]; therefore, removal of the developing fetus from the uterine environment could predispose a newborn to a heightened risk of blunted growth during the early childhood years. Indeed, Cutfield et al. [53] showed that very low birth weight (VLBW) and PB (≤32 weeks) in children aged 5 to 10 years corresponds to significantly lower plasma insulin-like growth factor 1 (IGF-1) and IGF-binding protein 3 (IGFBP-3) levels than those of full-term controls regardless of size for gestational age, which led the authors to conclude that PB contributes to low plasma IGF-1 and IGFBP-3 levels and the accompanying inability of children to attain their genetic height potential. Additionally, Kwinta et al. [55] demonstrated that serum IGF-1 levels were significantly lower in VLBW 7-year-olds born preterm than those of age- and sex-matched controls, and the authors attributed the smaller stature of the children born preterm to growth restriction associated with VLBW and low serum levels of IGF-1. PB-related alterations to IGF-1 and IGFBP-3 levels might also persist into adulthood. In a cohort of young adults, Verkauskiene et al. [56] found that adults born small for their gestational age had lower IGF-1/IGFBP-3 ratios and IGF-1 levels than adults born at weights appropriate for their gestational age. However, the age inclusion criterion for this study ranged from 32 to 42 weeks, including both individuals born premature and full term; therefore, alterations in IGF-1/IGFBP-3 ratios and IGF-1 levels could be attributed to size for gestational age independent of PB status. However, attainment of height potential and stature has been linked to VLBW independently of size for gestational age in young children, which suggests that VLBW associated with PB plays a role in mediating decreases in IGF-1 levels [53,55]. Stratification based on gestational age (e.g., 32–36 weeks, 36–40 weeks, 40–42 weeks) in future studies could elucidate our understanding of the role of premature status in long-term IGF-1 alterations. Additionally, intrauterine growth restriction—a process associated with PB [57]—has been linked to epigenetic changes in the IGF-1 gene of rats [58]. In this work, Fu et al. [58] showed increased DNA methylation in both P1 and P2 promoter regions in rats and corresponding decreases in serum IGF-1 levels in response to growth restriction, suggesting that alterations to the histone code of the IGF-1 gene could have long-lasting effects that blunt anabolic processes well into adulthood. Contrarily, Kantake et al. [59] showed reduced DNA methylation in the P2 promoter region of the IGF-1 gene in infants born preterm (<32 weeks’ gestational age) that could reflect an adaptation to growth restriction in utero. While the cause(s) of the discrepancies in IGF-1 methylation in rats and infants born preterm is not fully apparent, they could represent species-specific responses to growth restriction. Unfortunately, IGF-1 levels in the study by Kantake et al. were not measured [59], so the extent to which IGF-1 levels were influenced by epigenetic modification to the P2 promoter region in infants born preterm is unknown.

Since growth hormone (GH) largely controls the secretion of IGF-1, it is not surprising that exogenous use of both GH and IGF-1 has been explored to augment skeletal muscle hypertrophy and strength—a treatment strategy that could potentially benefit adults born prematurely, given their propensity for lower muscle mass [60]. Dong et al. [61] showed that when used in combination with gene therapy, IGF-1 therapy promotes skeletal muscle hypertrophy and improved contractile function in mice suffering from peripheral arterial disease, while Lynch et al. [62] demonstrated that IGF-1 treatment increases force-producing capabilities and skeletal muscle mass in dystrophic mice. In humans, recombinant human growth hormone (rHGH) supplementation has also increased lower body muscle strength in healthy 50-year-old men [63]. Moreover, rHGH supplementation in elderly men (aged 65–82 years) can significantly increase lean mass when combined with strength training [64]. These data suggest that using exogenous GH and IGF-1 can elicit beneficial changes to muscle size, strength, and contractile function in both mice and humans; however, the efficacy of such a strategy in adults born prematurely has not been explored. Moreover, characterizing the relationship between PB and long-term changes to IGF-1 is difficult due to a lack of longitudinal studies on the topic, so IGF-1 levels may normalize over time while other factors exert a more substantive effect on skeletal muscle hypertrophy potential and overall muscle function in adults born prematurely. Regardless, low IGF-1 levels in infants born preterm and developing children and epigenetic alterations to the IGF-1 gene because of growth restriction in utero suggest PB-related alterations to IGF-1 levels are possible. Therefore, longitudinal studies demonstrating a relationship between PB status and altered IGF-1 levels are needed to determine if IGF-1 or rHGH therapy would be viable options for increasing muscle mass and improving overall function in adults born prematurely.

## 3. Impaired Amino Acid Signaling and Protein Synthesis

Infants born prematurely show significantly lower weight and length growth rates than those of infants born at full term in the first three months after birth [65]; thus, nutritional interventions that include intravenous amino acid supplementation have been employed immediately after birth to accelerate fat-free mass accretion [66,67]. Unlike insulin, essential amino acids—especially leucine—stimulate the mTOR pathway through their interactions with Ras-related GTP-binding proteins (Rags), thereby activating muscle protein synthesis and fat-free mass accretion [68]. Indeed, protein balance and body weight increases have been attributed to amino acid supplementation (as much as 4.5g/kg/day in some cases) in infants born prematurely [69,70,71]. For example, using a two-compartment model of body composition (i.e., fat mass, lean mass), Salas et al. [66] demonstrated that 50 days of daily enteral protein intake increases fat-free mass and body weight in infants born extremely preterm (25–28 weeks’ gestational age). The results of this study could be interpreted to mean that increases in skeletal muscle were proportionate to increases in other tissues that comprise lean mass; however, given that lean mass comprises numerous tissues (e.g., organs, bone, muscle, and water), increases in body weight and/or lean mass reflect only broad changes that may or may not be indicative of increased skeletal muscle mass [72]. Therefore, increases in body weight and lean mass that occur after enteral protein treatment could be attributed to an overall increase in total body protein accretion, with a large proportion of the changes occurring in lean tissues (e.g., bone, organs) other than skeletal muscle, while increases in body weight could reflect a combination of increased total body protein accretion and increased calorie consumption (i.e., subjects in the supplementation group consumed more calories than the control group). Regarding the latter scenario, Cooke et al. [73] showed that when calories are equated, high-protein formula elicits greater weight gain than low-protein formula in infants born preterm. Unfortunately, this study did not include measures of lean mass, but the results suggest that lean mass was likely increased to a larger degree in the high-protein group, given that calories were equated [73]. Regardless, evidence of protein-supplement-induced changes in the skeletal muscle of infants born preterm is lacking, while data from animal models show that amino acid sensitivity in skeletal muscle is blunted in response to protein supplementation [17,18,74]. Since adults born prematurely have lower lean mass than adults born at full term [60], this could signify an inherent limitation in individuals born prematurely that affects their ability to increase skeletal muscle mass into adulthood.

Rudar and Naberhuis [75] showed that preterm neonatal pigs display lower mTOR·RagA and mTOR·RagC complex abundance in longissimus dorsi muscle than full-term controls 2 h after enteral amino acid administration, which is further supported by the findings of Rudar et al. [18], who showed no change in mTOR·RagA and mTOR·RagC complex abundance in the gastrocnemius muscle of preterm pigs after leucine supplementation. Briefly, the amino acids arginine and leucine stimulate the amino acid-sensing proteins stress response protein 2 (Sestrin2) and cytosolic arginine sensor for mTORC1 subunit 1 (CASTOR1), respectively, causing inhibition of GATOR1 via GATOR2, simultaneous activation of Rag proteins (i.e., GTP loading of Rag A/B; GDP loading of Rag C/D), and eventual mTOR activation [17,76]. Moreover, preterm neonatal pigs show downstream effects after amino acid administration, evidenced by lower eukaryotic initiation factor 4E/G-binding protein (eIF4E·eIF4G) abundance than full-term controls [75]. Altogether, preterm pigs showed a 28% lower increase in protein synthesis in the longissimus dorsi muscle after amino acid supplementation than full-term pigs; however, protein synthesis rates in the brain, liver, heart, and lung were not statistically different between groups [75]. Similar results were obtained by Naberhuis et al. [17], where amino acid administration elicited greater increases in liver and kidney mass but lower increases in longissimus dorsi mass in preterm pigs than in full-term pigs. Alterations in amino acid signaling were further demonstrated by Suryawan et al. [18], where the association between RagA/mTOR and RagC/mTOR in longissimus dorsi muscle was significantly lower in preterm pigs than in full-term pigs after amino acid supplementation. Importantly, small Rag GTPases are responsible for translocating mTORC1 to the lysosomal membrane before its activation by Rag [77], so alterations to Rag proteins could disrupt mTOR translocation and subsequent protein synthesis. Thus, lowered muscle protein synthesis in preterm pigs could be related to a disruption in Rag activation. Interestingly, gestational age has not been shown to affect the dissociation of Sestrin2 from GATOR2, which suggests that prematurity blunts Rag activation via an alternative mechanism (e.g., prematurity-related alterations to folliculin that result in decreased GDP loading of Rag C/D) [17,75]. Notwithstanding the lack of a clear mechanistic explanation for Rag protein alterations in premature pigs, the evidence shows poor Rag protein abundance and corresponding decreases in mTOR activation and muscle protein synthesis in response to amino acid supplementation. Whether or not these alterations are present in human infants born preterm, and if present, whether they continue throughout adulthood, is unknown; therefore, future research should focus on prematurity-related alterations to Rag proteins and the corresponding effect on hypertrophic potential.

## 4. Poorly Developed Mitochondria and Associated Inflammation

**Figure 1:**
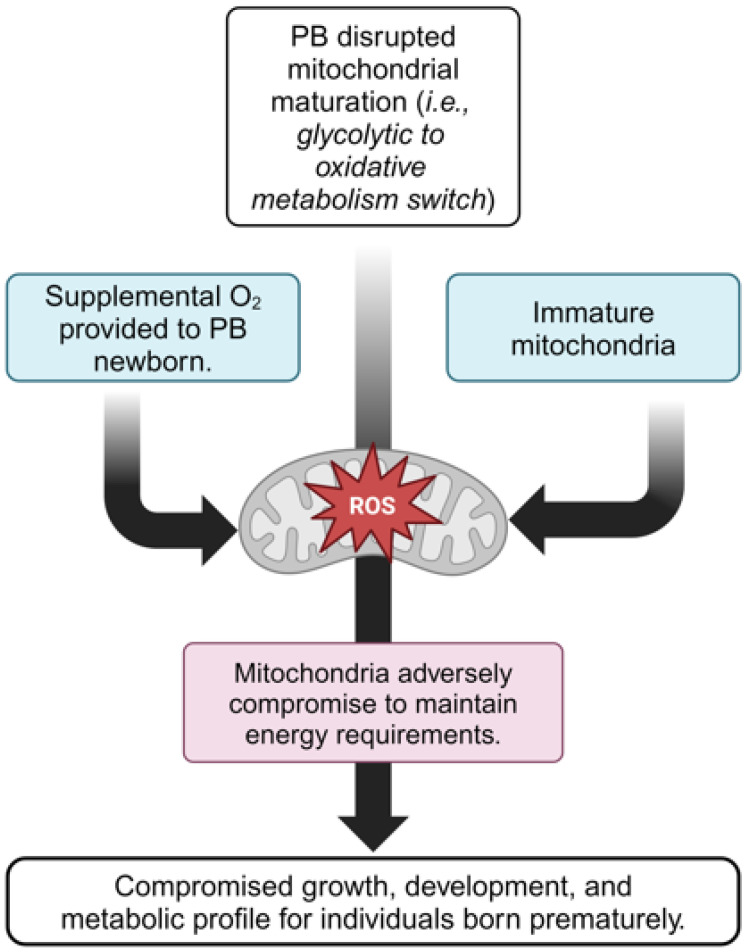
Illustration of the potential factors linking early-birth-inhibited mitochondrial maturation and a compromised metabolic profile in individuals born preterm (the illustration is the authors’ own work, created with Biorender.com, accessed on 4 April 2024).

Infants born preterm miss the metabolic shift characterized by an increased reliance on mitochondrial metabolism and decreased reliance on glycolysis, and approximately 90% of infants born very to extremely premature are provided supplemental oxygen to sustain life [78,79]. Thus, an infant born preterm may not have fully mature mitochondria across all cell types, and early exposure to oxygen could perpetuate dysfunction and increase disease risk. Indeed, exposure to excessive oxygen combined with immature mitochondria sets the stage for a vicious cycle of high oxidative stress (reactive oxygen species, ROS) and subsequent inflammation (Figure 1). Briefly, excessive and/or persistent ROS production can result in the activation of the transcription factor nuclear factor kappa-light-chain-enhancer of activated B cells (NF-κB), leading to increased transcription of inflammatory cytokines (e.g., interleukin-6 [IL-6], tumor necrosis factor-alpha [TNF-α], interleukin-1 beta [IL-1β], and interleukin-8 [IL-8]) [80] and concurrent inflammation that can activate the E3 ubiquitin ligase MuRF1, thereby increasing muscle protein degradation, inhibiting myofiber regeneration, and preventing late-stage myotube differentiation [81,82,83]. While it is unknown how skeletal muscle mitochondrial function changes from birth throughout life in those born very preterm, one study by Kumari et al. [84] found that mitochondrial dysfunction is present in peripheral blood mononuclear cells of young adults born preterm, signified by greater mitochondrial uncoupling, higher basal mitochondrial respiration, and higher maximal mitochondrial respiration than those born at full term. While this finding seemed counterintuitive, the authors speculated that the higher respiration phenomenon was a maladaptive response initiated by severe oxidative stress at birth, where, throughout life, respiration exceeds normal function as a protective mechanism against further excessive oxidative-stress-inducing exposures, a process known as mitohormesis [85]. Importantly, these mitochondrial respiration traits in young adults born preterm are not protective against disease, and, in an animal model of preterm birth (induced via hyperoxia at birth), those that developed higher mitochondrial respiration rates developed heart failure as adults, with the condition being more pronounced in males [86].

Given that preterm individuals already exhibit increased inflammatory cytokine expression [87], the added effect of hyperoxia could perpetuate inflammation. Along these lines, Deprez [60] showed that 3–10 days of exposure to hyperoxia leads to long-lasting effects in the hindlimb muscles of preterm rats. Specifically, they showed increased phosphorylation of the NF-κB subunit p65 (Ser536)—which can cause increased transcriptional activity depending on the physiological context [88]—in addition to increased CD43+ (neutrophil marker) and CD68+ (pan-macrophage marker) cells [60]. Moreover, Deprez et al. [60] demonstrated that muscle atrophy and fiber type shifting (from type I or IIa oxidative to type IIb fast glycolytic) are carried into adulthood in hyperoxia-exposed preterm rats, which suggests that even brief hyperoxic exposures in neonates born preterm can have long-term consequences on skeletal muscle phenotype. The diminished proportion of type I fibers observed by Deprez et al. [60] in hyperoxia-exposed preterm rats is also consistent with the findings of Keens et al. [29], who showed that neonates born preterm (<37-week gestational age) possess a significantly lower proportion of type I muscle fibers in the ventilatory muscles than neonates born at full term and adults. While a lower proportion of type I fibers and a correspondingly high proportion of type IIx fibers can impair aerobic performance [89,90], given the comparatively low mitochondrial density of type II fibers [91], contracting type II fibers also produce 2- to 3-fold more ROS (H_2_O_2_; hydrogen peroxide) than contracting type I fibers [92]; thus, greater type II fiber expression can be yet another source of inflammation that further limits skeletal muscle growth and impairs muscle function. While inflammatory cytokines indirectly blunt skeletal muscle hypertrophy through increased skeletal muscle catalysis, they can also affect hypertrophic signaling directly via inhibition of mTOR signaling [93]. Indeed, TNF-α-induced hyperexpression of the ubiquitin ligases muscle RING finger protein-1 (MuRF1) and F-box only protein 32 (MAFbx) has been shown to blunt Akt, mTOR, p70S6K1, and 4E-BP1 expression [93]. Therefore, ROS-mediated increases in inflammation have the cumulative effect of increasing skeletal muscle protein degradation while simultaneously limiting skeletal muscle hypertrophy. However, it is essential to note that more studies are needed to establish a clear link between early-life inflammation and impaired anabolic potential in individuals born preterm, but the evidence presented justifies further research in this area. Moreover, the effect of exercise on these maladaptive mitochondrial traits in individuals born preterm is currently unknown. Regardless, mitochondria are the cells’ major energy source for organ development and functional aspects where energy demand is high. Thus, it is plausible that oxidative-stress-induced disruptions (i.e., from supplemental oxygen) combined with underdeveloped mitochondria are significant factors contributing to impaired skeletal muscle growth and development in individuals born preterm.

Because mitochondria are a primary source of oxidative stress and therefore plausibly play an outsized role in mediating skeletal muscle impairments in those born prematurely, establishing early-life (i.e., infancy) interventions to offset and/or prevent impaired skeletal muscle development throughout critical developmental years is an important consideration. Thus, effective early-life interventions may include those targeted to enhance the function of mitochondria and their ability to respond to excessive oxidative stress when exposed to high supplemental oxygen. However, with the known high incidence of young adults born preterm [1], interventions aimed toward improving their mitochondrial function must not be ignored as this dysfunction has been linked to early-life onset of metabolic disease [10].

## 5. Epigenetic Changes

As previously discussed, PB is associated with an increased risk of diabetes. Among the numerous metabolic intermediates that influence chromatin modifications, NAD^+^ and acetyl-CoA are among the most well characterized due to their association with diseases such as diabetes [31,94,95] and cancer [96]. While its effect on gene expression is indirect (i.e., it exerts its effect on proteins rather than histones), increased NAD^+^ is associated with increases in autophagy and concomitant reductions in both inflammatory cytokine production and oxidative stress via sirtuin 1 (SIRT1)-mediated deacetylation of the forkhead box protein transcription factor (FOXO1) [97,98]. Contrarily, decreased NAD^+^, as shown in diabetic patients [31,99], could lead to reduced SIRT1- and SIRT2-mediated deacetylase activity and corresponding reductions in autophagy [100,101]. On the other hand, increases in acetyl-CoA, a metabolic intermediate that serves as the primary acetyl donor for histone acetyltransferases (HATs) [102], can cause increased histone acetylation that favors a reduction in autophagy [103,104]. Moreover, increases in HAT activity (i.e., increased histone acetylation) promote greater expression of proinflammatory cytokines because of HAT-NF-κB interactions [105]. Since NAD^+^ and acetyl-CoA induce changes in gene expression and concomitant inflammation through their indirect interactions (i.e., via sirtuins and HATs) with NF-κB, it seems reasonable to infer that PB-related diabetes could lead to an overall increase in inflammation that blunts skeletal muscle hypertrophic signaling through mechanisms such as TNF-α-mediated MuRF1 and MAFbx hyperexpression [93]. Thus, increases in ROS (due to the poor antioxidant capabilities of type II muscle fibers) [60], decreases in NAD^+^, and increases in acetyl-CoA could amplify the production of inflammatory cytokines [101,103,104], albeit by separate mechanisms, which could lead to impairments in skeletal muscle hypertrophy.

DNA methylation, which leads to gene repression or inhibition of transcription factor binding via the addition of a methyl group to a cytosine nucleotide in DNA (i.e., CpG site) [106], differs based on birth weight [107]. Moreover, differential methylation patterns at birth can also be retained until at least age 18 [108]; therefore, a possible consequence of PB-related low birth weight could be long-term DNA methylation patterns that affect several physical characteristics, including skeletal muscle hypertrophic potential. Interestingly, Relton et al. [109] showed that DNA methylation of the alkaline phosphatase (ALPL) gene locus at birth is associated with height at age 9, suggesting that early-life DNA methylation can partially mediate growth potential. Regardless, our understanding of the epigenetic regulation of PB-related adverse health effects is still unclear, which precludes any definitive conclusions regarding metabolite- or methylation-mediated alterations to hypertrophic signaling. However, while sparse, the evidence presented does appear to suggest that PB-related alterations to NAD^+^ and acetyl-CoA abundance could lead to impaired hypertrophic signaling because of protein and/or chromatin modifications that promote increased expression of proinflammatory cytokines, while low-birth-weight-associated changes in DNA methylation might also play a role in blunting anabolic potential in individuals born preterm. Long-term studies with large sample sizes are needed to understand better the relationship between early-life differential gene expression and long-term muscle growth potential. Importantly, the known mitochondrial maladaptation and consequential inflammatory response could contribute to and/or substantiate epigenetic alterations linked to preterm birth.

## 6. Conclusions

**Figure 2:**
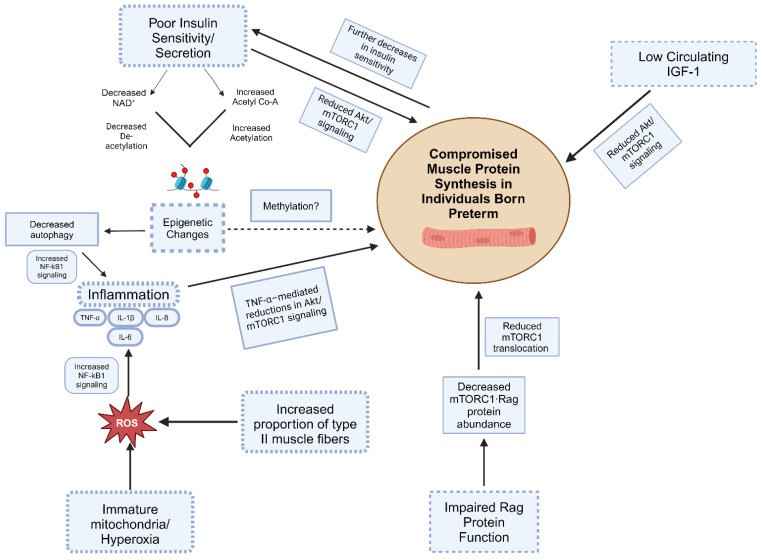
Summary illustration of potential mechanisms of PB-induced adverse effects on skeletal muscle health in individuals born preterm.

In this review, we provided evidence from both human and animal studies that suggests a potential link between PB and impaired skeletal muscle growth in individuals born preterm (Summarized in Figure 2). Specifically, we demonstrated how features associated with PB, such as diabetes, could not only blunt skeletal muscle growth independently via reduced insulin signaling but also potentially influence other physiological processes (i.e., metabolite-mediated changes in autophagy gene expression) known to mediate skeletal muscle growth (i.e., increases in inflammation via reductions in autophagy). Thus, if PB attenuates skeletal muscle growth over the long term, the causes are likely multifactorial and interconnected. However, we recognize that evidence of long-term impairments to skeletal muscle growth in individuals born preterm is lacking, as long-term studies with large sample sizes are needed to confirm a causal relationship between PB and sustained alterations to skeletal muscle growth.

## 7. Future Directions

Future research should focus on elucidating the mechanisms that govern potential PB-mediated alterations to skeletal muscle hypertrophy. As previously mentioned, skeletal muscle anabolic potential depends on numerous factors, and those highlighted in this review likely represent only a few of many factors. Further, hormone treatment (e.g., IGF-1, rHGH), supplementation (e.g., vitamin D), and resistance training can also potentially minimize PB-induced skeletal muscle growth impairments. Thus, further research is needed to assess the effectiveness of these interventions on mechanisms linked to preterm-birth-induced effects on skeletal muscle health impairments. Identifying such target mechanisms early in the life of individuals born prematurely and establishing interventions to enhance skeletal muscle growth and development are critical for preventing the early onset of metabolic diseases and mortality in this population.
